# Lymphopenia as an Independent Marker of Disease Activity in Children with Systemic Lupus Erythematosus

**DOI:** 10.3390/children12040486

**Published:** 2025-04-10

**Authors:** Gartika Sapartini, Reni Ghrahani, Budi Setiabudiawan

**Affiliations:** 1 Department of Child Health, Faculty of Medicine, Universitas Padjadjaran/Dr. Hasan Sadikin General Hospital, Bandung 40161, Indonesia; reni.ghrahani@unpad.ac.id (R.G.); budi.setiabudiawan@unpad.ac.id (B.S.); 2 Faculty of Medicine, President University, Bekasi 17550, Indonesia

**Keywords:** anti-dsDNA, clinical manifestation, disease activity, lymphopenia, systemic lupus erythematosus

## Abstract

**Background**: Lymphopenia is associated with disease activity in adult patients with systemic lupus erythematosus (SLE), but no similar studies exist among children. Furthermore, lymphopenia has only been used as a parameter of disease activity in the SLE disease activity index (SLEDAI), but not as an independent marker. **Objectives**: This study aimed to ascertain lymphopenia as an independent marker related to disease activity in children with SLE. **Methods**: This was a retrospective cohort study on patients newly diagnosed with SLE. The data were collected from January 2009 to March 2017, including clinical manifestations, complete blood counts, anti-dsDNA, and Mexican-SLEDAI (MEX-SLEDAI) scores. Statistical analysis was performed using the Chi-square test, Student’s *t*-test, and ROC curve analysis. **Results:** A total of 103 patients, aged from 12 to 18 years, participated in the study. Of these, 58 patients (56.3%) exhibited lymphopenia. The most commonly observed clinical manifestations in the lymphopenia group included nephritis (72.4%), hypertension (24.1%), and leukopenia (36.2%), with *p* < 0.05. Furthermore, neuropsychiatric SLE was found exclusively in the lymphopenia group. A negative correlation was observed between lymphocyte counts and anti-dsDNA levels (r = −0.24), as well as between lymphocyte counts and the MEX-SLEDAI score (r = −0.63, with *p* < 0.05). The receiver operating characteristic (ROC) curve indicated that a lymphocyte count with a cut-off point of ≤1738/mm^3^ is significant for predicting anti-dsDNA reactivity. **Conclusions**: Lymphopenia is significantly correlated with higher anti-dsDNA levels and increased disease activity, potentially serving as an independent marker of disease activity in children with SLE. However, further research is needed.

## 1. Introduction

Systemic lupus erythematosus (SLE) is a chronic autoimmune disease characterized by the excessive production of autoantibodies against self-antigens and the formation of immune complexes that cause damage to several organ systems, with a broad spectrum of clinical manifestations [1,2]. Among all SLE patients, 20% are often diagnosed in childhood, and the course of childhood onset is more progressive compared to adults [3]. The activity at the beginning and during the disease is more severe, causing higher morbidity and mortality in childhood-onset than adult-onset SLE [2]. Meanwhile, the mortality rate ranges from 5.7% to 31.2% among Southeast Asian children and is often caused by infections in the first few years, followed by end-stage renal disease and the severe exacerbations of lupus [2,4]. The exact prevalence of SLE in Indonesia remains undetermined. A study in Yogyakarta indicated that the frequency of SLE was 10.6% in 2015, increasing to 35.2% from January to September 2018 [5].

Assessment of disease activity is important for patient management, including changes in medical conditions and the impact of the disease on patients [6]. Systemic lupus erythematosus disease activity is identified through systematic clinical assessment, serological tests and the measurement of the disease activity index. Meanwhile, serum autoantibodies, namely antibodies to double-stranded DNA (anti-dsDNA), are widely used for measuring disease activity and diagnosing flares in SLE, and their serum levels often correlate with lupus activity [6]. Several disease activity indexes are used to assess the disease activity of SLE. One such index that has been validated for children is the MEX-SLEDAI. This index is a modified version of the SLEDAI, developed by Guzman et al., to reduce the cost of laboratory tests typically included in the SLEDAI. This cost reduction is particularly important in developing countries, where testing for anti-dsDNA antibodies and C3 complement levels may be limited. The MEX-SLEDAI has demonstrated a strong correlation with the SLEDAI-2K, making it a suitable option for assessing lupus disease activity. Additionally, it is less expensive than the SLEDAI-2K. Therefore, the MEX-SLEDAI serves as a reasonable alternative to the SLEDAI 2K for evaluating disease activity in lupus patients worldwide, particularly in clinical or research settings with limited resources [7].

Certain clinical manifestations are used as a parameter for assessing the MEX-SLEDAI score, such as mucocutaneous, renal, and musculoskeletal disorders; fever; fatigue; vasculitis; serositis; neuropsychiatric SLE; and hematological disorders, including hemolysis, thrombocytopenia, leukopenia, and lymphopenia [7]. Meanwhile, lymphopenia is one of the most common clinical manifestations of SLE, and it is often detected in approximately two-thirds of lupus patients on initial diagnosis and more than 90% during the disease [8,9,10]. Lymphopenia is associated with certain clinical manifestations, such as nephritis, neurologic involvement, and arthritis [11,12]. The clinical applications of lymphopenia are not only limited to SLE diagnosis, which is one of the hematological criteria in the American College of Rheumatology/ACR 1997, but they also serve as one of the parameters used to assess disease activity in the MEX-SLEDAI [6,11]. 

Several studies stated that lymphopenia is associated with disease activity in adult patients with SLE [13,14,15], but there are no similar studies among children. The monitoring of SLE disease activity with an anti-dsDNA test in developing countries, such as Indonesia, is relatively expensive and only available in a few healthcare facilities. In contrast, white blood cell and lymphocyte counts are carried out as part of routine hematology tests, available in almost every healthcare facility. 

Lymphopenia, which is measured by the lymphocyte count, has only been used as a parameter of disease activity in the MEX-SLEDAI score, but not as an independent marker of SLE disease activity. Nevertheless, lymphocyte count is a good and inexpensive alternative marker to determine SLE disease activity [11]. Therefore, this study aims to ascertain lymphopenia as an independent marker related to disease activity, by identifying the association between lymphopenia and clinical manifestations, anti-dsDNA, and disease activity in children with SLE.

## 2. Materials and Methods

This retrospective cohort study investigated patients who were newly diagnosed with SLE according to the ACR 1997 criteria, with ages ranging from 0 to 18 years. The research was conducted at the Child Health Department of Hasan Sadikin General Hospital in Bandung, West Java, Indonesia. Data were collected from medical records between February 2017 and March 2017, at the time of diagnosis, following the acquisition of written consent from the patients’ parents. The collected data included clinical manifestations, white blood cell and differential counts, anti-dsDNA levels, and the disease activity index as assessed by the MEX-SLEDAI score. Patients with alternative causes of lymphopenia, such as viral hepatitis, tuberculosis, or a prior history of immunosuppressive treatment, were excluded from the study.

The patients were divided into two groups based on their lymphocyte count. The first group, known as the lymphopenia group, included patients with a lymphocyte count of fewer than 1500 cells/mm^3^. The second group, called the non-lymphopenia group, comprised those with a lymphocyte count greater than 1500 cells/mm^3^. Additionally, the level of disease activity was evaluated using the MEX-SLEDAI score, which is a modified and validated version of the SLEDAI. This modification simplifies the assessment process while ensuring reliability in measuring disease activity in SLE [16]. 

The MEX-SLEDAI score is a simplified version of the original SLEDAI score, developed by Mexican researchers in 1992. This scoring system has a sensitivity of 87.5% and a specificity of 100%. In the MEX-SLEDAI score, fatigue and lymphopenia were added, while lupus headache and visual disturbances were removed. Unlike the original SLEDAI score, the MEX-SLEDAI does not include laboratory test results, such as complement levels and anti-dsDNA antibodies. The MEX-SLEDAI score evaluates various conditions related to SLE, including the following: (1) Neurological disorders, seizures, psychosis, organic brain syndrome, and cerebral nerve disorders. (2) Cardiovascular disorders. (3) Renal disorders, kidney function is evaluated based on the presence of casts, hematuria, and proteinuria. (4) Hematological conditions, including hemolysis and thrombocytopenia; (5) Dermatological conditions, such as new rashes, alopecia, and mucocutaneous disorders. (6) Serositis, which refers to pleuritis, pericarditis, and peritonitis. (7) Additional factors, such as lymphopenia, vasculitis, arthritis, and relative myositis. It is important to note that lymphopenia is included in the parameters for the MEX-SLEDAI score but is excluded from the final score calculation. The final score is derived from various variables grouped by target organ, with a total range from 0 to 32. A score of 5 or higher indicates active disease, and a higher score reflects more severe SLE disease activity [16,17].

### Sample Size and Statistical Analysis

The sample size for this study was determined based on the research objectives, using a confidence level of 95% and a power of 80%. To analyze the association between lymphopenia and clinical symptoms in children with SLE, a sample size formula was utilized to assess the differences between the two proportions.

$$n=\frac{{\left(Z_{\propto /2}\sqrt{2PQ}+Z_{\beta }\sqrt{P_{1}Q_{1}+P_{2}Q_{2}}\right)}^{2}}{{\left(P_{1}-P_{2}\right)}^{2}}$$

The formula for determining the sample size indicated that 25 participants were needed for each group. 

For the second and third objectives, specifically examining the correlation between lymphopenia and anti-dsDNA levels in children with SLE, as well as the correlation between lymphopenia and disease activity in these children, the sample size formula was used to evaluate the difference between two means.

$$n=2{\left(\frac{{(Z}_{\frac{\propto }{2}}+Z_{\beta })SD}{{\bar{X}}_{1}-{\bar{X}}_{2}}\right)}^{2}$$

According to the formula above, we have *n* = 33 and *n* = 11 for each group, respectively. 

Statistical analyses were conducted using the Chi-square test, Student’s *t*-test, and Spearman rank correlation, with significance defined at *p* < 0.05. ROC curve analyses were performed to determine the cut-off point for lymphocyte counts, utilizing SPSS Windows version 24. 

## 3. Results

A total of 103 patients, consisting of 58 patients (56.3%) with lymphopenia and 45 (43.7%) without lymphopenia, participated in this study. Most of the patients were 12–18 years old, and the female-to-male ratio was 16:1. The characteristics of SLE patients are shown in Table 1, as follows.

The mean age of the lymphopenia group was 12.67 ± 2.76 years, which was higher than that of the non-lymphopenia group, 11.3 ± 3.17 years. Additionally, the median age for the lymphopenia group was 13.09 years (range: 6.58–18.75), compared to 11.67 years (range: 3.42–18.70) for the non-lymphopenia group. The youngest participant in the non-lymphopenia group was 3.42 years old.

Table 2 shows that the main clinical manifestations in the lymphopenia group, compared to the non-lymphopenia group, include nephritis (72.4% vs. 13.3%), hypertension (24.1% vs. 6.7%), and leukopenia (36.2% vs. 6.7%), with a *p*-value less than 0.05 for all comparisons. Additionally, neuropsychiatric SLE was found exclusively in the lymphopenia group. These findings suggest that children with SLE and lymphopenia face significantly higher disease risks, as follows: they are 10.86 times more likely to experience nephritis, 3.62 times more likely to develop hypertension, and 5.43 times more likely to have leukopenia, compared to children with SLE without lymphopenia. 

**Figure 1:**
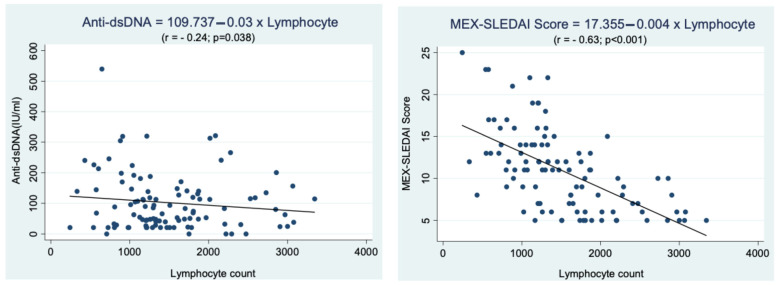
Correlation between lymphocyte count and anti-dsDNA levels and disease activity (MEX-SLEDAI score) in children with SLE.

The regression analysis was carried out to determine the correlation between lymphocyte count, anti-dsDNA levels, and disease activity (Figure 1). There was a negative correlation between lymphocyte count and anti-dsDNA levels, with a weak correlation strength (r = −0.24, *p* < 0.05), which implies that the lower the lymphocyte count, the higher the anti-dsDNA levels in children with SLE. Similarly, there was a negative correlation between lymphocyte count and MEX-SLEDAI score, with a strong correlation strength (r = −0.63, *p* < 0.05), meaning that the lower the lymphocyte count, the higher the MEX-SLEDAI score. 

The correlation between lymphopenia and disease activity in children with SLE was explained using the median MEX-SLEDAI score. This score was higher (*p* < 0.05) in the lymphopenia group [13 (IQR 5–25)], compared to the non-lymphopenia group [7 (IQR 5–15)]. 

**Figure 2:**
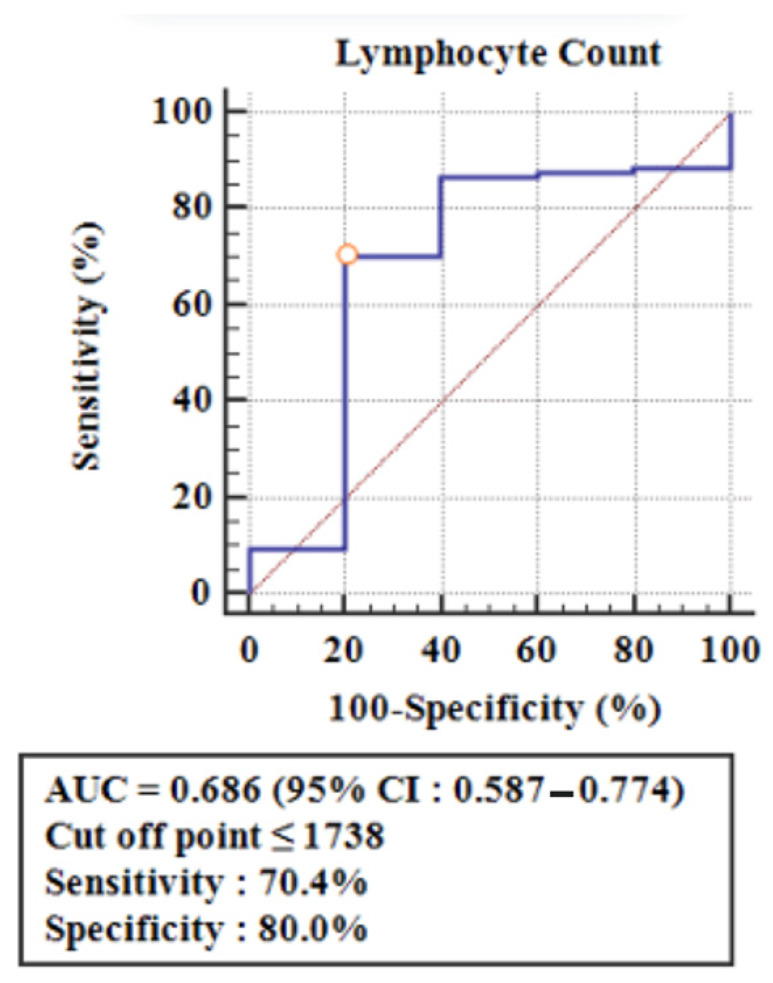
ROC curve to determine the cut-off point for the lymphocyte number as a predictor of anti-dsDNA reactivity.

Calculations were performed using the ROC curves, to determine the cut-off point for the number of lymphocytes capable of causing anti-dsDNA reactivity (Figure 2). The cut-off point ≤ 1738 lymphocytes/mm^3^ had a sensitivity of 70.4% (95% CI = 60.3–79.2) and a specificity of 80% (95% CI = 28.4–99.5) for predicting anti-dsDNA reactivity (AUC = 0.686, 95% CI = 0.587–0.774), and these results were statistically significant (Table 3). Table 3 also shows a positive predictive value (PPV) of 98.6%, meaning that when the number of lymphocytes is ≤1738/mm^3^, then the anti-dsDNA level of most children with SLE is reactive (true positive), and only a small part is non-reactive (false positive). In contrast, the negative predictive value (NPV) was 12.1%, which implies that when the lymphocyte count is >1738/mm^3^, then the anti-dsDNA levels of a few children with SLE are non-reactive (true negative), while the majority are reactive (false negative). Finally, this study showed a sensitivity of 70.4%, specificity of 80%, accuracy of 68%, PPV of 98.6%, and NPV of 12.1% (Table 3).

## 4. Discussion

The results showed that lymphopenia is associated with certain clinical manifestations in children with SLE. Furthermore, there was a correlation between lymphopenia and anti-dsDNA levels and the MEX-SLEDAI score, indicating disease activity in children with SLE. 

Lymphopenia is a clinical feature frequently observed in SLE and is one of the hematological criteria for its diagnosis, according to ACR 1997. Among the 103 patients studied, the lymphopenia group included 58 patients (56.3%), while the non-lymphopenia group consisted of 45 patients (43.7%). The outcomes for those with lymphopenia in this cohort were lower than the results reported by Vila et al. (64.6%), Faddah et al., Merayo et al., and Ng et al., where approximately two-thirds (66.7%) of SLE patients at their initial diagnosis exhibited lymphopenia [1,9,10,11]. Conversely, our findings indicated a higher rate compared to Rivero et al., who found lymphopenia in 75 out of 158 (47.5%) active and untreated SLE patients [12]. Lymphopenia is commonly associated with SLE and is particularly prevalent in patients with active or severe disease [18].

The average age of patients in the lymphopenia group at the time of diagnosis was 12 years and 8 months. This finding is consistent with that of Weiss et al., who reported a mean age of 12 years for pediatric-onset SLE, and Brunner et al., who observed a range of 12 to 14 years. However, this average is higher than the results reported by Ghrahani et al., at 10.5 years, and Evalina et al., at 10.25 years [18,19,20,21]. In contrast, the average age of the non-lymphopenia group was lower, at 11 years and 3.5 months. Both Brunner et al. and Levy et al. indicated that pediatric-onset SLE is infrequently observed in children under 5 years of age [2,19]. In this study, only two patients were younger than 5, specifically 3 years and 5 months and 4 years and 2 months old. Systemic lupus erythematosus is predominantly found in post-pubertal females [18], which aligns with the findings of this study that showed a predominance in the 12–18-year age range. The male-to-female ratio for LES was 1:16, which is higher than the ratios reported by Weiss et al., at 1:9; Ghrahani et al., at 1:10.6; and Evalina, at 1:11 [18,20,21].

Multiple and varying clinical manifestations might be found during SLE diagnosis, depending on the affected organs. The major clinical manifestation of SLE associated with significant lymphopenia is lupus nephritis. These results are consistent with Faddah et al. and Sobhy et al., who reported that the only significant clinical manifestation of SLE associated with lymphopenia is lupus nephritis [1,22]. Similarly, previous studies found that lymphopenia in patients with SLE is associated and might be a promising marker for kidney involvement [11,22]. This was further explained by Nakabayashi et al., who stated that 80% of nephritis patients with active SLE often have high anti-T cell antibodies, and the levels are 30–40% higher than in other glomerulonephritis cases [23].

Based on these results, lymphopenia is significantly associated with neuropsychiatric SLE. This is consistent with Rivero et al., who reported that lymphopenia is associated with neurologic involvement and arthritis, as well as with Yavuz et al., who showed an association between lymphopenia and neurologic disease [12,24]. Another study found that severe lymphopenia is associated with neuropsychiatric SLE [25]. This association is caused by the binding of anti-lymphocyte antibodies to a variety of target antigens. In addition to binding to lymphocytes, anti-lymphocyte antibodies also bind to neurons, namely via the anti-ribosomal P-protein. This protein binds to the antigen on the surface of T lymphocytes to induce apoptosis and cross-reacts with neurons [26]. Anti-lymphocyte antibodies that react with neurons are often pathogenic in neuropsychiatric SLE [27].

Lymphopenia was significantly associated with leukopenia, but not hemolytic anemia and thrombocytopenia, as reported by Vila et al. and Yu et al. [11,25]. Another clinical manifestation associated with lymphopenia is hypertension; however, the pathophysiological mechanism underlying the development of hypertension remains unclear [28]. Furthermore, the results showed that the clinical manifestation of nephritis was more common in the lymphopenia group compared to the non-lymphopenia group (72.4% vs. 13.3%). In patients with lupus nephritis, there is a decrease in the glomerular filtration rate due to vasoconstriction, causing renal tubular dysfunction, which leads to hypertension [29]. However, this has not been reported by previous studies and therefore needs further research.

The examination of anti-dsDNA is one of the parameters used to monitor disease activity in SLE [13]. These levels are assessed at the time of diagnosis and are continuously monitored throughout the progression of the disease. Elevated anti-dsDNA levels indicate active SLE [18]. Unfortunately, the anti-dsDNA test presents some limitations and is not available in every healthcare facility in developing countries. A previous study found that lymphopenia was associated with increased levels of anti-dsDNA antibodies in early SLE [11]. Additionally, Yu et al. reported a significant association between lymphopenia and anti-dsDNA antibodies at diagnosis and during flare-ups of the disease [25]. In this study, we investigate the correlation between lymphocyte counts and anti-dsDNA levels—a relationship that has not been previously reported. Our results indicate a negative correlation between lymphopenia and anti-dsDNA levels (r = −0.24), suggesting that lower lymphocyte counts are associated with higher levels of anti-dsDNA antibodies in children with SLE.

This study found a significant relationship between lymphopenia and disease activity in children with SLE. Specifically, there was a negative correlation between lymphocyte count and the MEX-SLEDAI score, with a correlation coefficient of r = −0.63 and a p-value of <0.05. This indicates that a lower lymphocyte count is associated with a higher MEX-SLEDAI score. Additionally, the median MEX-SLEDAI score in the lymphopenia group was 13 (range 5–25), which was significantly higher than the score in the non-lymphopenia group, which was 7 (range 5–15). These findings align with previous studies, such as that of Vila et al., who reported a strong association between moderate (500–999/mm^3^) to severe lymphopenia (<500/mm^3^) and increased disease activity. Similarly, Mirzayan et al. found that lymphopenia predicted more severe disease and exacerbations during a one-year follow-up in patients with SLE [11,13]. The increased T cell apoptosis observed in active SLE, along with high expression levels of membrane-bound and soluble Fas, may contribute to this phenomenon [1,30]. Furthermore, CD4+ and CD8+ T cells that express the CD28 molecule—a critical co-stimulatory signal for T cell activation—are often found to be decreased in the peripheral blood of SLE patients. CD28-mediated co-stimulation appears to influence T cell susceptibility to activation-induced cell death, which may play a role in lymphopenia [1,15]. These results suggest that lymphopenia is associated with higher disease activity and might serve as an independent predictor of severe disease. Thus, measuring lymphocyte counts can be considered a valuable marker related to SLE disease activity [24,31].

Lymphopenia is defined by a lymphocyte count of less than 1500/mm^3^, based on the criteria from ACR 1997, or a median count of 1.3 × 10^9^/L in patients with active SLE, as reported by Han et al. [32]. Lymphocyte counts were recorded only at the time of diagnosis. Receiver operating characteristic (ROC) curves were then used to identify the intersection point and differentiate the number of lymphocytes associated with anti-dsDNA reactivity. The cut-off point for lymphocyte count was established at ≤1738/mm^3^, which is higher than both the ACR 1997 criteria and the findings from Han et al. [32]. This cut-off demonstrated a sensitivity of 70.4%, a specificity of 80%, and an accuracy of 68% in predicting anti-dsDNA reactivity, with statistical significance. The positive predictive value (PPV) was found to be 98.6%, indicating that when the lymphocyte count is ≤1738/mm^3^, most anti-dsDNA levels are reactive, with only a small fraction being non-reactive or false positives. Therefore, if the lymphocyte count is ≤1738/mm^3^, it is likely that the anti-dsDNA level will be reactive. This finding suggests that lymphocyte count could serve as a potential marker for predicting anti-dsDNA reactivity in children with SLE, which is particularly useful for healthcare facilities that are unable to conduct anti-dsDNA tests. However, no previous studies have explored the correlation between lymphocyte counts and anti-dsDNA reactivity, highlighting the need for further research in this area.

Our study has several limitations. First, as with all retrospective cohort studies, our findings may be subject to information bias because they rely on pre-existing medical records. However, despite being retrospective, our study provides critical insights that could lay the groundwork for future prospective research. These insights include the identification of an inexpensive alternative marker for predicting SLE disease activity, specifically through lymphocyte counts. Future prospective studies are needed to confirm these findings. Second, we identified a cut-off value for lymphocyte count to predict anti-dsDNA reactivity. However, we acknowledge that external validation in independent cohorts is necessary before this threshold can be widely applied in clinical practice. Future multicenter studies with larger sample sizes and diverse populations are needed to evaluate its generalizability. Third, we recognize that lymphopenia is correlated to elevated serum interferon levels in SLE, indicating immune dysregulation. However, our study did not assess interferon levels, as they were not part of our current research objectives. Future research that incorporates interferon biomarkers could provide additional insights into the role of lymphopenia in disease activity. Finally, our study did not utilize the SLEDAI-2K due to budget constraints. It was conducted in a hospital with limited access to laboratory tests, specifically complement tests. Consequently, the MEX-SLEDAI was the most feasible option for assessing disease activity while maintaining clinical relevance.

## 5. Conclusions

In conclusion, this study showed a significant association between lymphopenia and the more severe clinical manifestations of SLE, including nephritis, hypertension, and neuropsychiatric SLE. It also found a correlation with higher levels of anti-dsDNA antibodies and increased disease activity. Lymphocyte counts of ≤1738/mm^3^ can serve as a useful threshold for predicting anti-dsDNA reactivity in children with SLE, showing good sensitivity (70.4%) and specificity (80%). Moreover, the PPV of 98.6% indicates that most patients with this lymphocyte count are likely to have reactive anti-dsDNA levels, making it a reliable marker for disease activity, particularly in healthcare facilities that cannot perform anti-dsDNA tests. Therefore, lymphopenia may be considered an independent marker of SLE disease activity, although further research is needed to validate these findings.

## Figures and Tables

**Table 1:** Characteristics of the patients.

Characteristics	Lymphopenia (*n* = 58)	Non-Lymphopenia (*n* = 45)	Total (*n* = 103)
Age (years)	12.67 ± 2.76	11.3 ± 3.17	
2–6 years	0 (0%)	2 (100%)	2 (100%)
6–12 years	23 (47.9%)	25 (52.1%)	48 (100%)
12–18 years	35 (66%)	18 (34%)	53 (100%)
Gender			
Male	4 (66.7%)	2 (33.3%)	6 (100%)
Female	54 (55.7%)	43 (44.3%)	97 (100%)

**Table 2:** Association between lymphopenia and clinical manifestation in children with SLE.

Clinical Manifestation	Lymphopenia (*n* = 58)	Non lymphopenia (*n* = 45)	*p*-Value	Prevalence Ratio (CI 95%)
Constitutional				
Fever	21 (36.2%)	11 (24.4%)	0.201	1.48 (0.80–2.74)
Fatigue	19 (32.8%)	19 (42.2%)	0.323	0.70 (0.47–1.28)
Weight loss	12 (20.7%)	12 (26.7%)	0.477	0.70 (0.39–1.56)
Mucocutaneous				
Malar rash	35 (60.3%)	26 (57.8%)	0.631	1.04 (0.75–1.45)
Discoid rash	15 (25.9%)	15 (33.3%)	0.408	0.78 (0.43–1.41)
Photosensitivity	17 (29.3%)	13 (28.9%)	0.963	1.01 (0.55–1.86)
Oral ulcer	23 (39.7%)	16 (35.6%)	0.671	1.12 (0.67–1.85)
Alopecia	25 (43.1%)	26 (57.8%)	0.140	0.75 (0.51–1.10)
Joints				
Arthralgia	22 (37.9%)	18 (40%)	0.831	0.95 (0.50–1.54)
Arthritis	17 (29.3%)	16 (35.6%)	0.500	0.82 (0.47–1.44)
Cardiopulmonary				
Pleural effusion	13 (22.4%)	5 (11.1%)	0.134	2.02 (0.78–5.24)
Pericardial effusion	12 (20.7%)	13 (28.9%)	0.336	0.72 (0.36–1.42)
Valve anomaly	3 (5.2%)	2 (4.4%)	1.000 *	1.16 (0.20–6.67)
Pulmonary hypertension	3 (5.2%)	1 (2.2%)	0.410 *	2.33 (0.25–21.63)
Dilated cardiomyopathy	4 (6.9%)	1 (2.2%)	0.303 *	3.10 (0.36–26.82)
Vascular				
Raynaud’s phenomenon	1 (1.7%)	1 (2.2%)	1.000 *	0.70 (0.05–12.07)
Gangrene	0	0		-
Kidney				
Nephritis	42 (72.4%)	6 (13.3%)	<0.001 ^#^	10.86 (3.60–32.79)
Hypertension	14 (24.1%)	3 (6.7%)	0.018 ^#^	3.62 (1.11–11.84)
Neuropsychiatry				
Psychosis and seizure	16 (27.6%)	0	<0.001 ^#^	-
Hematology				
Hemolytic anemia	41 (70.7%)	33 (73.3%)	0.767	0.96 (0.76–1.23)
Leukopenia	21 (36.2%)	3 (6.7%)	<0.001 ^#^	5.43 (1.73–17.07)
Thrombocytopenia	6 (10.3%)	3 (6.7%)	0.728 *	1.55 (0.41–5.87)

Note: The *p*-value was calculated based on the Chi-squared test; * Fisher’s exact test. ^#^ *p*-value < 0.05, statistically significant; PR (95% CI), prevalence ratio and 95% confidence interval.

**Table 3:** Category of lymphocyte count used as the cut-off point of anti-dsDNA reactivity.

Anti-dsDNA Group			
Category	Reactive *n* (%)	Non-Reactive (%)	Total *n* (%)
Lymphocyte ≤ 1738	69 (70.4%)	1 (20%)	70 (68%)
Lymphocyte > 1738	29 (29.6%)	4 (80%)	33 (32%)
Total	98 (100%)	5 (100%)	103 (100%)

## Data Availability

The data will be made available on the study’s primary site. Please contact the corresponding author for future access due to privacy and ethical reasons.
